# Influenza activity in Saint Joseph, Missouri 1910-1923: Evidence for an early wave of the 1918 pandemic

**DOI:** 10.1371/currents.RRN1287

**Published:** 2011-11-17

**Authors:** Brian L. Hoffman

**Affiliations:** Department of Natural and Physical Sciences, Park University, Parkville, Missouri

## Abstract

While the 1918/1919 H1N1 influenza pandemic is widely recognized as a “worst-case scenario” for the emergence of a new influenza strain, relatively little is known about the origin of the responsible virus and its pattern of spread. Most studies of this virus in the United States rely on temporally and spatially aggregated data. Location-specific studies of the impact of the 1918 pandemic strain in the United States have been confined primarily to large cities on the East Coast or West Coast. In this study, data on pneumonia and influenza fatalities from 1910-1923 have been extracted from death certificates for Saint Joseph, Missouri, a typical mid-sized city in the central United States. An increase in pneumonia and influenza mortality was noted starting in the 1915/1916 influenza season. Initially, increased mortality was observed in infants and the elderly. In February 1918, an age-shift typical of pandemic strains of virus was seen, as the burden of mortality shifted to young adults, a characteristic of the 1918 pandemic virus. These results provide one of the first confirmations of the existence of a “herald wave” of influenza activity in the United States prior to the recognized start of the H1N1 pandemic in Spring 1918. This study is one of very few that measures the impact of 1918/1919 influenza in a particular location in the central United States.

## Introduction

The 1918-1920 H1N1 influenza A pandemic caused unprecedented morbidity and mortality on a global basis.  Within the span of two years, about 1/3 of the world's population had sickened from influenza; in excess of 675 000 people died from "Spanish influenza" in the United States and as many as 50-100 million people died worldwide [1] [2] [3] [4].  Since then, three more pandemics have occurred, with far less morbidity and mortality: the 1957/1958 H2N2 "Asian influenza"; the 1968/1969 H3N2 "Hong Kong influenza"; and the 2009/2010 swine-derived H1N1pdm influenza that emerged in Mexico and the United States [5] [6] [7] [8].  Analysis of the three pandemics from the 20th century show that five features characterize influenza pandemics: 1) emergence of a novel subtype of influenza virus; 2) shift of peak death rates to younger age groups; 3) multiple waves of pandemic virus activity; 4) increased transmissibility compared to seasonal viruses; and 5) variation in impact across different geographic regions [9]. 

Virological evidence collected during the last three pandemics has contributed greatly to our knowledge of the bases of their virulence and evolution.  However, the 1918/1919 pandemic occurred before the cause of influenza was known, and virological evidence of influenza A evolution prior to 1930 is severely limited. Seroarchaeology first indicated the cause of the 1918/1919 pandemic as an H1N1 influenza A virus [10].  This was recently confirmed with isolation of H1N1 influenza virus RNA sequences from preserved tissues of soldiers in the United States that had died in the severe autumn wave of pandemic influenza [A/South Carolina/1/18 (H1N1) and A/New York/1/18 (H1N1)] and an Inuit woman who died in November 1918 at Brevig Mission, Alaska [A/Brevig Mission/1/18(H1N1), abbreviated BM/18] [11] [12].

The "signature age-shift" of pandemic viruses was evident in 1918 [13] [14].  During prepandemic years, the age-specific pneumonia and influenza (P&I) mortality curve was U-shaped, with relatively high mortality rates in infants and adults over-65 years of age.  During the 1918/1919 pandemic year, the mortality curve took on a W-shape, with a peak in mortality in young adults as well as in infants and older adults [15].  Age-shifts have also been noted during 1918/1919 by comparing excess mortality rates in older adult populations and younger populations,  in which the burden of excess mortality shifts to the younger age groups [14] [16].The reasons for the peak in mortality among young adults is not well understood, but studies with recombinant viruses in murine and primate systems suggest that the robust immune system of a healthy young adult may initiate a "cytokine storm" in response to BM/18, resulting in massive cell death in infected tissues [17] [18] [19] [20].  Immune experience may have played a role in bending the age-specific P&I mortality curve into a W-shape, assuming that the very young are totally naive to H1N1 influenza viruses, those older than 30 years having been exposed to H1N1 viruses prior to 1890 and retaining some immunity to that subtype of influenza virus coupled with the elderly having reduced levels of immunity because of underlying illness or senescence [21] [22].  The low levels of mortality seen in school-aged children can be explained by the phenomenon of the "honeymoon period" during which a general lack of mortality due to serious illness is seen in this age group [21].

The 1918/1919 pandemic rippled across the globe in three recognized waves .  In the United States, these were experienced as a relatively mild spring wave, a severe autumn wave roughly from September - December 1918, and a mild spring 1919 wave [23].  The first wave was generally noted in late spring - early summer in Europe [24].  Many authors have suggested that February 1918 influenza outbreaks in Haskell County, Kansas followed by a March influenza epidemic at Camp Funston, Fort Riley, Kansas represent the origin of the first wave in the United States [25] [26] [27].  Influenza then reportedly spread through United States military camps, to nearby urban centers and then Europe with influenza-infected soldiers and sailors of the American Expeditionary Force and US Navy.  This argument has been weakened by demonstration of pandemic activity in New York City starting in February 1918 [16].  Only one other study has shown this early wave in the United States previously (in Louisville, Kentucky) using archaeo-epidemiological methods [26].  Differential retention of immunity to a subtype of influenza virus after infection may explain this multiwave pattern of pandemic virus spread [22].

Transmissiblity in viruses that cause seasonal epidemic influenza varies with the number of susceptible members of the population.  During the initial wave of 1918/1919 pandemic influenza, the reproductive number appears to have been between 2.0 - 5.4 [24] [28].  The reproductive number dropped to 1.2-2.1 during the autumn wave in countries that had experienced the earlier wave [16] [24] [28] [29].  This is just one line of evidence that indicates that infection with an earlier, less lethal form of pandemic influenza virus may immunize a substantial portion of the population, mitigating the effect of a second wave of infection [30] [31]

Excess mortality during the 1918/1919 H1N1 pandemic varied greatly by geographic region.  In the United States, excess mortality from all causes is estimated to have been 0.39% of the population.  The total excess mortality rate was highly variable from state to state: Missouri had a 0.39% rate; Colorado 1.0%; Michigan 0.26%; and Pennsylvania 0.81%.  Global rates also showed a wide range of variation.  International excess all cause mortality rates ranged from 4.39% in India to 0.2% in Denmark.  Within India, the rates ranged from 7.8% in the Central Provinces and Berar to 2.1% in Burma [4] [24]. 

This study used death certificate information to explore trends in influenza mortality in Saint Joseph, Missouri from 1910-1923.  The data shows a trend of growing mortality among infants from the 1915/1916 season to beyond the 1918/1919 pandemic year.  Mortality among older adults jumped to the highest levels during 1915/1916 and then decreased through the pandemic year.  A shift in burden of excess mortality to younger age groups is demonstrated to have occurred twice in Saint Joseph, Missouri during the time period 1910-1923.  At least two waves of pandemic activity occurred, a mild wave in February-May 1918 and a severe wave from October 1918 - May 1919.  Following the pandemic, several shifts in age-specific mortality were observed, likely the result of intra-subtypic reassortments that helped generate the seasonal H1N1 influenza strain.  These data further extend the idea that a "herald wave" of pandemic activity sprang from widely separated geographic locations in the United States early in 1918, prior to the generally recognized outbreaks in Kansas.

## Materials and Methods

### Study setting

Saint Joseph is a city in northwest Missouri approximately 50 miles north of downtown Kansas City.  The 1910 city population was 77 408, growing slightly in 1920 and 1930 to populations of 77 948 and 77 939 in an area of 13.7  mi^2^.  Other  locations related to the pandemic are nearby, including Camp Funston at Fort Riley, Kansas and Haskell County, Kansas about 200 km and 600 km to the southwest, respectively.

**Figure 1:**
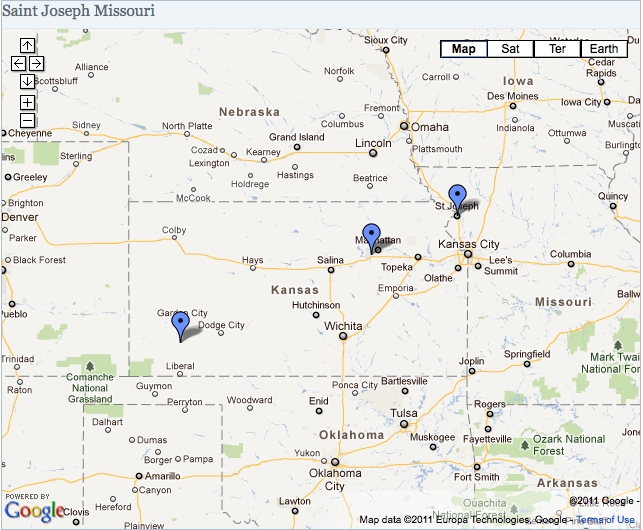
Location of Saint Joseph, Missouri and local sites related to the 1918/1919 influenza pandemic

### Mortality statistics

Death certificates in Missouri more than 50 years old were obtained online from the Missouri State Archives' Missouri Death Certificates 1910-1959 database.  P&I fatalities were identified from death certificates that listed influenza, flu, grippe, capillary bronchitis, heliotrope cyanosis or any form of pneumonia as a primary or contributing cause of death.  All study subjects died within the city limits and also resided or contracted the disease at a Saint Joseph city street address.  P&I deaths were stratified into ten age-groups for further analysis: under-1 year; 1-4 years; 5-9 years; 10-14 years; 15-19 years; 20-24 years; 25-34 years; 35-44 years; 45-64 years; and 65 years and older.  Population numbers were linearly interpolated from the 1910, 1920 and 1930 population census for the purpose of determining mortality rates.  A stratified random sample of census enumeration sheets was used to determine the population age structure for 1920 and 1930.

Excess mortality was calculated as the difference between mortality rate in a defined period of study and the expected, or baseline rate.  This study uses the five year P&I average mortality rate from influenza seasons between 1910 and 1914 as the baseline for calculating excess deaths.  This period of time was chosen because influenza seasons appeared to be fairly uniform and death records were available starting in 1910.  Several researchers have noted that increased P&I mortality was widespread in the United States during the 1915/1916, 1916/1917 and 1918/1919 influenza seasons.  These seasons were left out of the baseline because they potentially represent early waves of pandemic virus activity [2] [6]. 

Epidemic periods were determined by calculating the mean number of monthly P&I deaths during the 1910-1914 seasons and the 95% confidence interval for this data.  Epidemic periods were defined as being two or more consecutive months in which the number of P&I deaths exceeded the upper limit of the 95% confidence interval.

## Results

### Pneumonia and influenza activity in Saint Joseph 1910-1923

The 1910-1914 influenza seasons typically showed peaks in P&I deaths in January-March (Fig. 2).  During these seasons, there were approximately 112 P&I deaths per influenza season.  In the 1915-1917 seasons, P&I deaths rose to 191 per season with peak months showing nearly double the number of deaths.  The 1918/1919 season resulted in 530 P&I deaths and October-December of that season were the three worst months for P&I mortality since death records were reliably kept in St. Joseph.  This death rate was rivaled only by that seen in February 1920.   The following season (1920/1921) was mild, with P&I mortality at its lowest since 1910.

**Figure:**
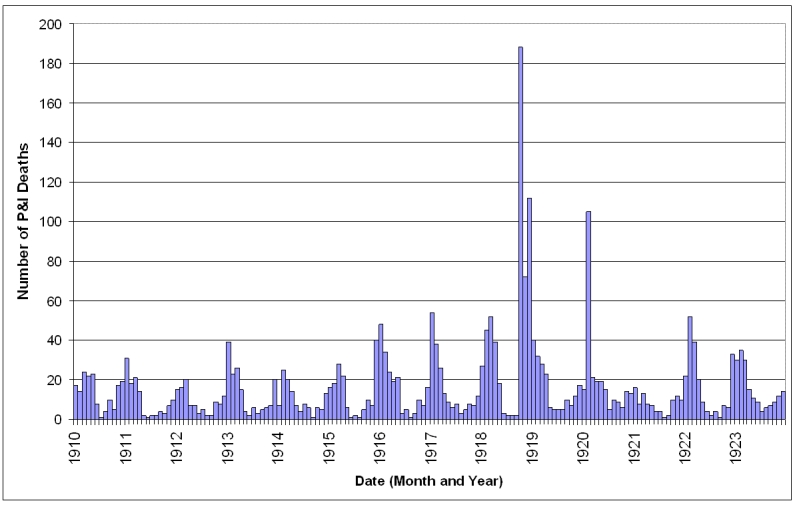
**Figure 2: Monthly pneumonia and influenza deaths in Saint Joseph, Missouri January 1910 - December 1923. ** 

The increase in P&I deaths from the 1915 influenza season apparent in Figure 2 was investigated further.  The expected number of deaths for an influenza season was calculated from the 1910-1914 seasons and the mean subtracted from the observed number of deaths in each season from 1910-1922.  As has been noted elsewhere, the 1911/1912 season was a rather mild season [16] [23], with negative excess mortality.  The other seasons in the baseline period had slight positive excess mortality, but were very close to expected numbers.  In the 1915/1916 influenza season mortality increased significantly and, with the exception of the 1921/1922 season, remained elevated much above baseline throughout the study period.  As expected, excess mortality was at a peak during the 1918/1919 pandemic season.  The 1919/1920 and 1917/1918 seasons ranked as the second and third worst seasons, in terms of excess mortality, causing about 25% of the excess deaths as in the pandemic year. 

**Figure:**
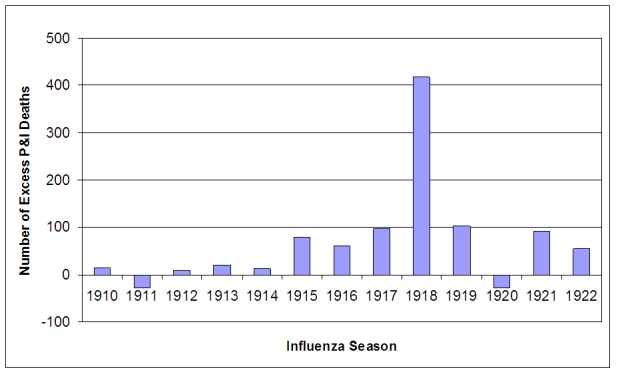
**Figure 3:  Number of excess P&I deaths for influenza seasons (October - May starting in indicated year) 1910-1922.**  The baseline number of deaths (1910-1914 mean) was subtracted from total number of deaths in each influenza season. 

Monthly P&I mortality data were utilized to find periods of epidemic P&I activity (Figure 4).  The upper 95% confidence interval limit for the 1910-1914 influenza seasons P&I deaths was set as the epidemic threshold.  Seven periods of time were identified as local P&I epidemics by the criterion of being consecutive months during which P&I deaths exceeded the epidemic threshold [16]: December 1915 - February 1916; January - February 1917; February - May 1918; October 1918 - April 1919; May - June 1920; August - September 1920; February - March 1922; and February - March 1923.  February 1920 was also included as an epidemic month because P&I mortality was at a level exceeded only by that observed in October and December 1918.

**Figure:**
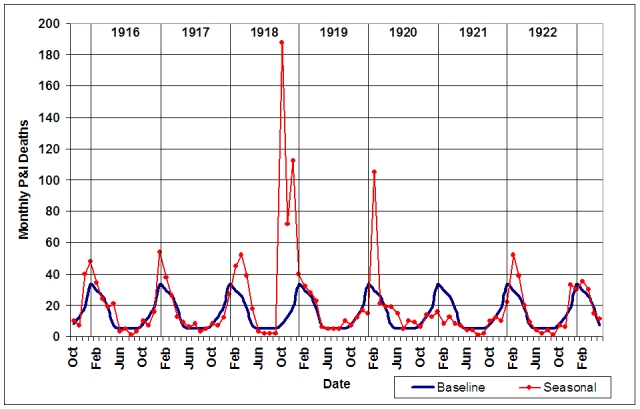
**Figure 4: Monthly P&I mortality from October 1915-May 1923 (red line) compared to upper 95% confidence interval limit (blue line) for 1910-1914 influenza seasons.**  Epidemic periods are defined as periods of time when consecutive months exceed the upper 95% confidence interval limit for the seasonal mean. 

### The W-Shaped Pandemic Season Curve

The mean seasonal P&I age-specific mortality curve for 1910-1914 exhibits the classic U-shape of interpandemic seasons.  The highest mortality rates were seen in the under 1 year and 65 years and older age groups during these years, with mortality being lowest in the young adult (15-44 year old) age groups.  During the 1918/1919 influenza season, the mortality curve shifted to the W-shape that has been well documented for the "Spanish influenza".  A dramatic shift in age pattern occurred in which overall mortality was high not only at both ends of the age spectrum, but also in young adults.  The only age group for which mortality was lower than baseline was the adults 65 years old and over.

**Figure:**
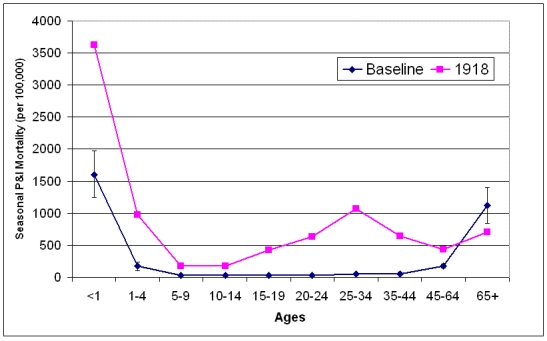
**Figure 5: Baseline all-ages seasonal P&I mortality for influenza seasons (October-May starting in indicated year) 1910-1914 and 1918.  **Error bars for baseline years represent the 95% confidence interval for mean seasonal P&I mortality.

### Seasonal Excess P&I Mortality 1915-1922

Age specific excess P&I mortality was calculated for each influenza season (October - May) from 1915-1922 (figures 6 and 7). Increased prepandemic virulence of influenza viruses from 1915 is in evidence across all age ranges.  With the exception of the 1-4 year age group in 1916/1917, in which mortality remained essentially at 1915/1916 levels, mortality increased each year in every under-65 year age group.  Mortality in the 65 year and over age group showed a unique pattern, rising sharply to a peak in the 1915/1916 season and decreasing each year through the pandemic season.

**Figure:**
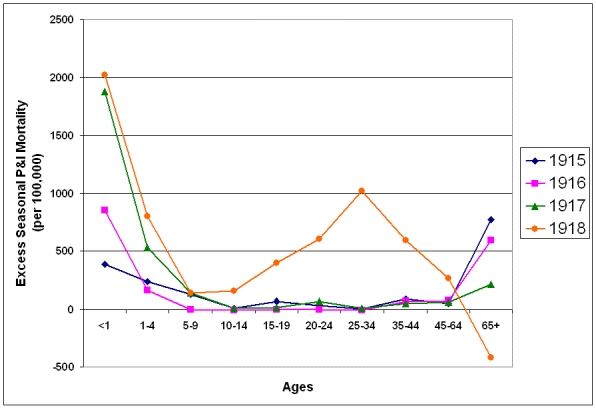
**Figure 6:  Seasonal excess P&I mortality for the 1915-1918 influenza seasons (October-May starting in indicated year).**

 After the pandemic year, P&I mortality fell towards prepandemic levels (figure 7).  In the 1919/1920 and 1921/1922 seasons, only a slight W-shape is apparent in the age-specific excess mortality curve, showing a slightly higher than normal mortality for the young adult age-groups, as well as the under 1 year and 65 and over age groups.  The 1920/1921 influenza season was mild in terms of excess deaths (figure 2).  In fact, all age groups except the 1-4 year age group had lower than normal mortality, giving the curve an upside-down U-shape.  The proportion of excess deaths represented by the under 65 year age groups also dropped towards levels more characteristic of interpandemic years (Table 1).

**Figure:**
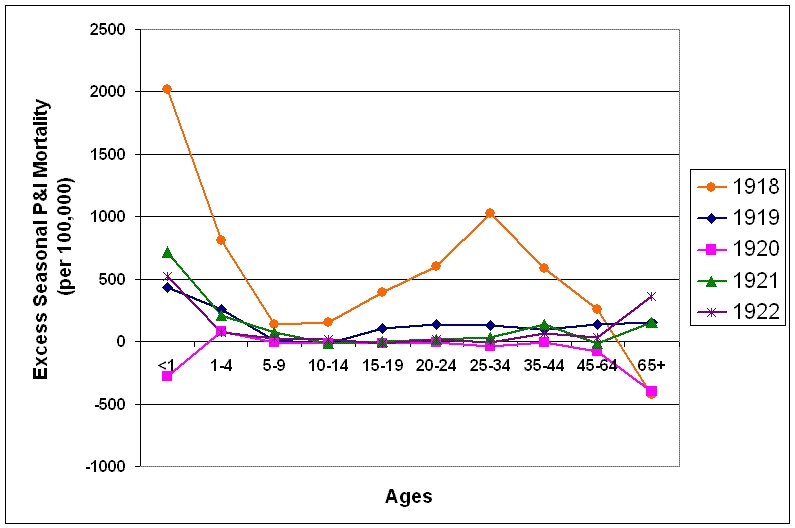
**Figure 7:  Seasonal excess P&I mortality for the 1918-1922 influenza seasons (October-May starting in the indicated year).**

A change in character of influenza viruses during the 1917/1918 season is apparent when the risk ratio of dying from P&I in those >65 years old when compared to those <65 are examined (Table 1).  Risk of P&I death shifted to the younger age groups during this season, shrinking to 25% of 1915/1916 levels.  This is due to an increase in mortality in the <65 year ages from the 1915-1918 influenza seasons, coupled with a decrease in mortality in the >65 year age group.  This trend reversed in the seasons following the pandemic season, another characteristic of influenza virus evolution [14].

**Table:** 

**Table 1. ** **Age-specific excess seasonal P&I mortality in St. Joseph, 1915-1923**					
	Excess P&I mortality rate/100,000				
Influenza season	All ages	Persons <65 years old	Persons >65 years old	Risk ratio >65:<65	Proportion (%) of excess deaths in persons <65 years old
1915/1916	103	68	836	12:1	63
1916/1917	79	49	682	14:1	59
1917/1918	124	114	321	3:1	88
1918/1919	534	562	0	0:1	100
1919/1920	131	122	308	3:1	88
1921/1922	79	60	363	6:1	76
1922/1923	70	39	605	16:1	53

### Excess mortality during epidemic periods 1915-1918

Three epidemic periods were identified from October 1915 - May 1918: December 1915-February 1916; January-February 1917 and February-May 1918.  Age-specific annual excess mortality rates were calculated to make values more comparable.  The epidemic period from December 1915 to February 1916 was characterized by a large increase in excess mortality in the >65 years age group.  During the following epidemic period in January-February 1916, excess mortality rose precipitously in the under-1 year age group while excess mortality in the oldest age group remained at the same level.  During the period February-May 1918, the W-shaped curve characteristic of the pandemic emerged, with highly elevated excess mortality in the under-5 year age groups and elevated excess mortality in the 20-24 year age group.  A dramatic shift in age-specific excess mortality patterns occurred during early 1918 as mortality in the >65 years age group dropped to 14% of 1915/1916 levels and mortality in the younger age groups rose.

The "signature age-shift" apparent during these three epidemic periods was further quantified using the risk ratio of dying from P&I by comparing mortality in those >65 years of age to those <65 years of age. While the risk ratio decreased from December 1915-February 1916 to January-February 1917, a major shift occurred in February 1918, when the risk ratio decreased nearly 65-fold from the previous month.  This ratio was 15-fold lower than the 1915/1916 epidemic period.  By March, all of the excess mortality had shifted to the <65 year age-groups, a pattern repeated during the 1918/1919 pandemic season.  By spring 1918, the proportion of all excess deaths seen in persons <65 years of age had more than doubled from 1915/1916 levels. 

**Figure:**
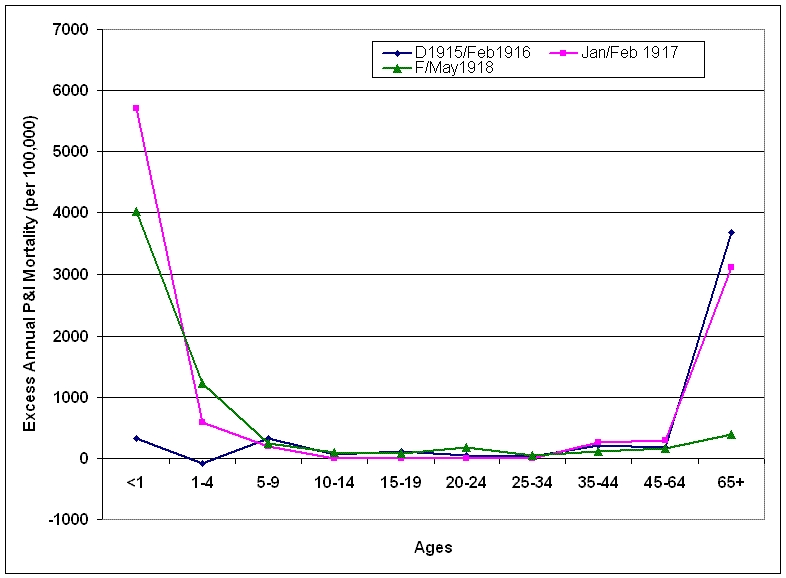
**Figure 8:  Age-specific excess P&I mortality during epidemic periods October 1915-May 1918.**  Epidemic periods were December 1915-February 1916 (D1915/Feb1916), January-February 1917 (Jan/Feb1917) and February-May 1918 (F/May1918).  Excess mortality rates are reported on an annual basis.

**Table:** 

**Table 2**. **Age-specific excess epidemic P&I mortality in St. Joseph, Oct 1915- May 1918**					
	Excess P&I mortality rate /100,000				
Epidemic period	All ages	Persons <65 years old	Persons >65 years old	Risk ratio >65:<65	Proportion (%) of excess deaths in persons <65 years old
Dec 1915-Feb 1916	72	30	920	31:1	41
Jan-Feb 1917	66	42	518	12:1	62
Jan 1918	8	1	133	133:1	43
Feb 1918	32	31	53	2:1	92
Mar 1918	37	42	0	0:1	100
Apr-May 1918	49	46	107	2:1	87

### Excess mortality during epidemic periods 1919-1923

The risk of P&I death remained elevated in under-65 age groups compared to the >65 year group for the four epidemic periods following the 1918/1919 pandemic season.  The excess mortality curve for the February 1920 epidemic took on a W-shape, as mortality increased greatly in adults 45 years and older.  During the next two epidemic periods in 1920, excess mortality dropped close to baseline levels in all age groups with the exception of children under 1 year of age.

**Figure:**
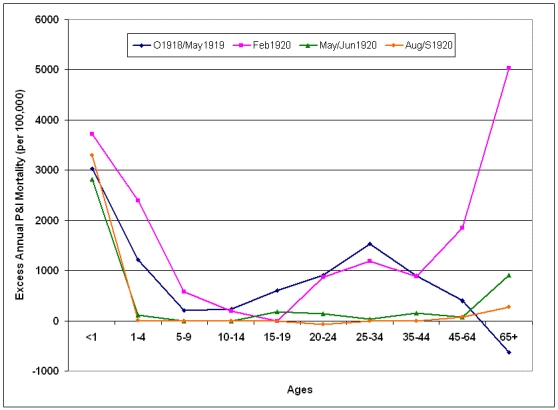
**Figure 9:  Age-specific excess annual P&I mortality during epidemic periods from October 1918-May 1921.**  Epidemic periods were October 1918 - May 1919 (O1918/May1919); February 1920 (Feb1920); May-June 1920 (May/Jun1920) and August-September 1920 (Aug/S1920).  Excess mortality rates are reported on an annual basis.

After the third of the post-pandemic epidemic periods, influenza activity declined in St. Joseph.  There were no epidemic periods during the 1920/1921 influenza seasons (Fig. 4) and there was negative overall excess mortality in that season (Fig. 7).  During February and March of the 1921/1922 influenza season, P&I activity again reached epidemic levels and a W-shaped age-specific excess curve emerged once more (Fig. 10).  Mortality was greatly elevated in the under-10 year old age groups, over 65 age groups and young adults, with a peak in the 35-44 year age group.  Excess mortality fell again toward baseline levels in most age groups during the February-March 1923 epidemic period, with the exception of the under 1 year and over 65 year age groups.  

**Figure:**
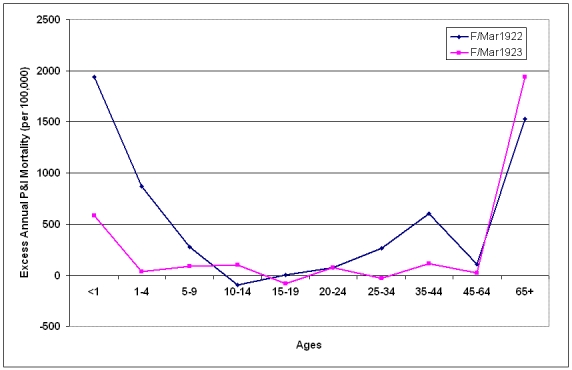
**Figure 10:  Age-specific excess annual P&I mortality during epidemic periods from October 1921-May 1923.**  Epidemic periods were February-March 1922 (F/Mar1922) and February-March 1923 (F/Mar1923).  Excess mortality rates are reported on an annual basis.

**Table:** 

**Table 3**. **Age-specific excess epidemic P&I mortality in St. Joseph, Oct 1919- May 1923**					
	Excess P&I mortality rate /100,000				
Epidemic period	All ages	Persons <65 years old	Persons >65 years old	Risk ratio >65:<65	Proportion (%) of excess deaths in persons <65 years old
Feb 1920	109	92	436	5:1	80
May-Jun 1920	32	26	126	5:1	79
Aug-Sep 1920	15	14	50	3:1	83
Feb-Mar 1922	62	47	314	7:1	73
Feb-Mar 1923	28	7	396	57:1	23

### Median age at Death

The median age of individuals who died of P&I was calculated for each season (Fig. 11) and epidemic period (Fig. 12)  from October 1910 - May 1923.  A distinct shift in mortality occurred during the 1917/1918 influenza season, with the median age at death dropping to half of the 1910-1916 seasonal levels.  Following the 1918/1919 pandemic year, the median age rose slowly, reaching pre-1917 levels during the 1922/1923 season.  An even larger shift is noticed when median age at death is plotted during P&I epidemic periods.  In February 1918, the median age for P&I deaths dropped to less than 10 years old, about 20% of the mean season median age of death from 1910-1914.  Median age of P&I deaths dropped again in March 1918, before rising to about 25 through the pandemic year.  The median age rose to near 1910-1914 levels during the February 1920 epidemic, then dropped again during the next two epidemics in May-June 1920 and August-September 1920.

**Figure:**
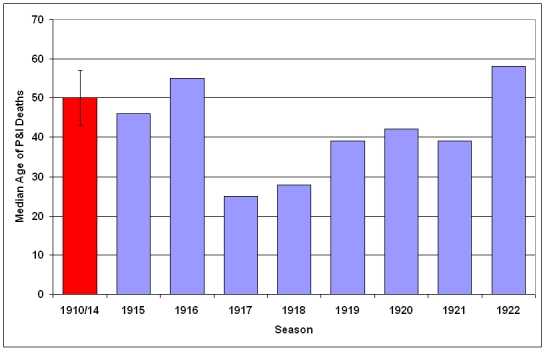
**Figure 11: Median age at death attributable to P&I for 1910-1922 influenza seasons (October-May starting in indicated year).**   Median age at death and 95% confidence interval for mean median age at death due to P&I  for years 1910-1914 is represented by red bar at the far left of the graph.

**Figure:**
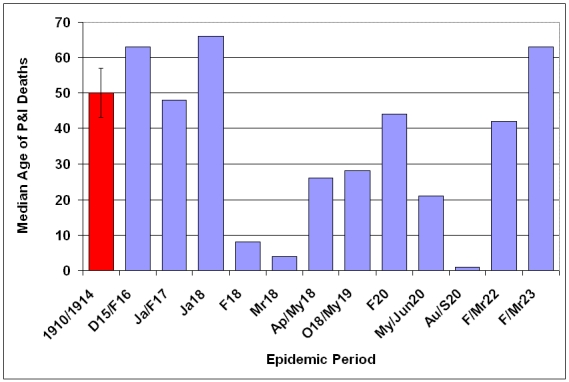
**Figure 12: Median age at death attributable to P&I for epidemic periods 1915-1923.**   Median age at death and 95% confidence interval for mean median age at death due to P&I  for years 1910-1914 is represented by red bar at the far left of the graph.

## Discussion

Were any of the influenza viruses that struck St. Joseph starting in 1915 related to the H1N1 pandemic strain of 1918/1919?  Lacking viral sequences isolated from infected patients at this location, it is difficult to know for certain.  However, the distinct age-shift observed from February 1918 suggest that a pandemic strain of influenza had reached St. Joseph by that date.

The epidemiological evidence in this study supports the hypothesis that a very virulent influenza virus exhibiting characteristics of a pandemic strain was active in the central United States during February 1918, which matches previous accounts of a serious influenza from Haskell County, Kansas in the same time frame.  Taken together with evidence that such a virus was also circulating in New York City in February 1918 [16], it seems likely that precursors to the pandemic strain were widely circulating in the United States prior to the March 1918 outbreak at Fort Riley, Kansas and subsequent spread through military camps and nearby urban centers.  This further contradicts the idea that central Kansas was the point of origin of the 1918 pandemic virus.

A significant rise in influenza-related mortality starting in December 1915 has previously been noted for the United States [32] [33].  Frost presented the possibility that this episode was potentially related to the 1918 pandemic [32], an idea that has been discussed by later authors, as well [2] [6].  Although evidence is mounting that pandemic viruses may be generated through multiple reassortment events occurring over several years [34] [35], there is no direct evidence of such an event in this population.  The age-distribution of influenza mortality in St. Joseph from 1915-1917 seems to indicate that the major influenza activity during this prepandemic period was due to seasonal influenza viruses, as the highest impact is evident in the extreme age-groups.  

The question of where the 1918 H1N1 pandemic influenza strain came from and when it arose is still very much up in the air.  It is clear that pandemic strains were circulating in the United States as early as February 1918.  The spring wave that followed, likely spreading the virus through military camps and adjoining urban areas, was most likely a result of the same virus.  A recent archeo-virological approach has provided evidence that the pandemic strain of 1918 was certainly present in the United States by May 1918 [36].  In a study of formalin-fixed, paraffin-embedded lung tissues from 68 soldiers from US Army training camps in the continental US who died from influenza and/or pneumonia between 11 May and 24 October 1918, H1N1 pandemic virus RNA sequences and/or antigens were shown to exist in 37 of these patients.  Four such patients died from May through August 1918, prior to the pandemic peak [36].

Although precursors to the pandemic virus may have circulated in human populations prior to the major autumn influenza wave of 1918, it is clear that a major change in the character of the virus occurred sometime between May and August 1918.  The origin of the changes that led to tremendous increases in mortality among young adults remains elusive.  Although it may not have been the sole origin of the spring 1918 epidemic, the Fort Riley epidemic and spread of influenza through the military complex likely was an amplifying event that culminated in intra-subtypic reassortments between viruses circulating in the United States and abroad.  Viruses with tremendous virulence, but ill-adapted to spread in human populations [37] [38] may have recombined with milder viral strains that spread easily between humans.  The sudden emergence of an influenza capable of generating a primary fulminating pneumonia in young adults in France, Sierra Leone and the United States in August 1918 may indeed point to recombination between viral strains from the US and Europe.

Phylogenetic evidence suggests that although the seasonal H1N1 virus that persisted following the 1918/1919 pandemic is related to the BM/1918 virus, it evolved through one or more intra-subtypic reassortments with other influenza viruses [39].  These studies indicate that only four of the eight segments of BM/1918 have remained in the seasonal H1N1 virus.  Intra-subtypic reassortants that introduce antigenic novelty larger than those expected due to point mutation have been implicated as the cause of the severe 1947 and 1951 epidemics [35]. Reassortment events may explain some of the larger shifts in age-specific mortality seen in St. Joseph after the 1918 pandemic, such as the large increases in mortality in older adults during the February 1920 epidemic and the February-March 1922 epidemic.  The appearance of a peak of mortality in the 35-44 year old age group also possibly points to a reassortment as the cause of the February-March 1922 epidemic period.

Relatively complete information about the emergence of pandemic strains of influenza A are only available for the four pandemics that occurred within the last 100 years.  Understanding the range of impacts that pandemic influenza strains have on populations depends on a complete analysis of the available data from these outbreaks.  Most of the data available for studying the 1918/1919 pandemic is temporally aggregated and lacks spatial referencing.  This severely limits the analysis of that data for a particular location.  The extraction of information from death certificates about individuals that died from P&I provides a unique tool to study the impact of the 1918/1919 pandemic on a typical mid-sized city in the central US.  The data are temporally discrete and spatially referenced, providing a way to analyze diffusion of the disease through the community, as well as measure differential impacts on a variety of subpopulations within the city.  Such a tool will help increase our knowledge of the 1918/1919 influenza pandemic as experienced in the central region of the US.  While several papers have described the pandemic on a national level or in cities of the East Coast or West Coast of the US, this paper represents one of the first analyses of the pandemic in the American Heartland.

### **Acknowledgments**

The author wishes to thank Albert Dusing, Scott Hageman, Ann Schultis, Brenda Royals, Zhilwan Rahim and an anonymous reviewer for providing helpful comments in the preparation of this paper.

### **Funding Information**

The author is grateful for support from Park University and the Park University Department of Natural and Physical Sciences for this project.

### **Competing Interests**

The author has declared that no competing interests exist.
